# A facile synthesis of PEGylated Cu_2_O@SiO_2_/MnO_2_ nanocomposite as efficient photo−Fenton−like catalysts for methylene blue treatment

**DOI:** 10.3389/fbioe.2022.1023090

**Published:** 2022-10-18

**Authors:** Mingzhou Wu, Shuqing He, Enna Ha, Junqing Hu, Shuangchen Ruan

**Affiliations:** ^1^ Key Laboratory of Optoelectronic Devices and Systems of Ministry of Education and Guangdong Province, Shenzhen Key Laboratory of Laser Engineering, College of Physics and Optoelectronic Engineering, Shenzhen University, Shenzhen, China; ^2^ College of Health Science and Environmental Engineering, Shenzhen Technology University, Shenzhen, China; ^3^ Sino-German College of Intelligent Manufacturing, Shenzhen Technology University, Shenzhen, China

**Keywords:** Cu_2_O@SiO_2_/MnO_2_−_PEG_, wastewater treatment, photocatalysis, photo−Fenton−like property, methylene blue

## Abstract

The removal of toxic organic dyes from wastewater has received much attention from the perspective of environmental protection. Metal oxides see wide use in pollutant degradation due to their chemical stability, low cost, and broader light absorption spectrum. In this work, a Cu_2_O−centered nanocomposite Cu_2_O@SiO_2_/MnO_2_−PEG with an average diameter of 52 nm was prepared for the first time *via* a wet chemical route. In addition, highly dispersed MnO_2_ particles and PEG modification were realized simultaneously in one step, meanwhile, Cu_2_O was successfully protected under a dense SiO_2_ shell against oxidation. The obtained Cu_2_O@SiO_2_/MnO_2_−PEG showed excellent and stable photo−Fenton−like catalytic activity, attributed to integration of visible light−responsive Cu_2_O and H_2_O_2_−responsive MnO_2_. A degradation rate of 92.5% and a rate constant of 0.086 min^−1^ were obtained for methylene blue (MB) degradation in the presence of H_2_O_2_ under visible light for 30 min. Additionally, large amounts of •OH and ^1^O_2_ species played active roles in MB degradation. Considering the enhanced degradation of MB, this stable composite provides an efficient catalytic system for the selective removal of organic contaminants in wastewater.

## 1 Introduction

Synthetic dyes and pigments like MB, Rhodamine B (RhB) and methyl orange (MO) used in the pharmaceutical, tannery, and textile industries are common sources of water pollution due to their complex aromatic molecular structure (Mohammadzadeh et al., 2020; Fan et al., 2021). This is especially true for textile manufacturing, as 10–50% of dye losses are discharged into the effluent (Uddin et al., 2021). Various physicochemical treatment strategies, including membrane filtration (Mohammed et al., 2020; Ni et al., 2022; Xiong et al., 2022), physical adsorption (Qiu et al., 2020; Huang et al., 2022), biological degradation (Deng et al., 2021), and advanced oxidation processes (AOPs) have been reported (Wang et al., 2021a; Li et al., 2022b). However, traditional treatments are hampered by recycling and economic costs. Using environmentally friendly, rapidly oxidative, and highly efficient pollutant elimination, photocatalysis and Fenton reactions are employed as common methods for pollutant elimination due to the oxidation activities of hydroxyl radicals (•OH) and superoxide anions (O_2_^•−^) (Zhang et al., 2021a; Wang et al., 2021b; Yang et al., 2022a).

Previous studies indicated that semiconductor materials such as metal oxides and transition metal sulfides are common photocatalysts. Metal−oxide−related photocatalysts underwent three generations, single−component materials, multiple−component materials, and solid substrate immobilized materials (Anwer et al., 2019; Ivanets et al., 2021). Materials with photo-sensitive reaction and Fenton-like reaction have been widely reported in the area of catalysis and bioapplication, such as metals (Yu et al., 2018), organics (Qiu et al., 2022), and metal-organic frameworks (Yu et al., 2021a; Liu et al., 2022; Yu et al., 2022). Cuprous oxide (Cu_2_O) is a promising p−type semiconductor with a 2.1 eV bandgap and displays a wide absorption band in the visible light region (Yang et al., 2016). Moreover, considering its inexpensive and convenient synthesis, abundant resources, and non−toxicity, Cu_2_O has been explored as an ideal candidate for photocatalysis (Wu et al., 2012). However, nanosized Cu_2_O particles readily oxidize in air or humid conditions, which significantly limits their application (Zhai et al., 2014). To stabilize Cu_2_O and improve its catalytic activities, reductive components have been utilized. For example, Wang et al. reported stable Cu_2_O nanoparticles supported on reduced graphene oxide (Cu_2_O/RGO), which showed excellent activity and recyclability towards Sonogashira cross−coupling of aryl halides with phenylacetylene and Ullmann coupling of phenols with aryl halides (Zhai et al., 2014; Wang et al., 2017a). Recently, a Ag−Cu_2_O composite film and a Ag/Cu_2_O heterojunction were applied in dye degradation reported by Yu et al. and Li et al., respectively. They illustrated that Ag could improve the photocatalytic performance by facilitating photoelectron transfer and accelerating the separation of photoelectron−holes in Cu_2_O. MB degradation rate of over 95.1% was obtained over Ag−Cu_2_O composite film (Yu et al., 2021b). Similarly, methyl orange (MO) was almost complete eliminated within 40 min under visible light irradiation using Ag/Cu_2_O (Li et al., 2022a). Moreover, hybrid Cu_2_O−Cu cubes exhibited visible−light−driven degradation against MO and rhodamine B (RhB) in the presence of H_2_O_2_ as a sacrificial scavenger. Due to the efficient separation of electron−hole pairs and improved charge transfer, Cu_2_O−Cu exhibits superior photocatalytic performance (Alp, 2021). However, the reductive component in composite materials is often sensitive to H_2_O_2_−conducted degradation systems.

The improvements of metal oxides related composite materials are commonly attributed to electron−hole separation facilitation, which promoted active species production (•OH, O_2_^•−^ etc.) during degradation (Hao et al., 2021). Could additional O_2_ improve the degradation efficiency? Herein, we sought to develop an ideal material functional in both O_2_ improvement and Cu_2_O protection. MnO_2_ is well known for O_2_ production from H_2_O_2_ via Fenton−like reaction for water treatment, biological antibacterial, and anticancer applications (Wang et al., 2018; Sun et al., 2021; Yang et al., 2021; Gemeay et al., 2008) reported polyaniline/MnO_2_ composites in the oxidative decolorization of organic dyes with H_2_O_2_. Based on H_2_O_2_ bubble generation over Fe_3_O_4_@MnO_2_, a removal efficiency of 99% was reached *via* advanced oxidation and adsorptive bubble separation (Kang et al., 2019). Recently, Jiao et al. synthesized MnO_2_ nanoparticle−loaded poly (amidoxime−hydroxamic acid)−modified microcrystalline cellulose (pAHA−MCC@MnO_2_). The birnessite−like MnO_2_ nanoparticles on the pAHA−MCC microrod surfaces played a vital role in MB due to advanced Fenton−like catalysis (Jiao et al., 2021). Therefore, combining Cu_2_O and MnO_2_ might develop an efficient catalyst for MB degradation in the presence of both visible light and H_2_O_2_.

In this work, a novel composite catalyst Cu_2_O@SiO_2_/MnO_2_−PEG was designed as photo−Fenton−like catalysts by introducing a highly dispersed ultrafine MnO_2_ and a dense SiO_2_ shell. This creative development sought to protect Cu_2_O from instability coupled with producing MnO_2_ from KMnO_4_ and PEG, which endow the sample with photo−Fenton−like catalytic activity. The synthetic procedure occurs at lower temperatures and with low energy consumption. The catalytic performance of Cu_2_O@SiO_2_/MnO_2_−PEG was subsequently evaluated by degrading MB dye in the presence of H_2_O_2_ under visible light irradiation. Furthermore, the mechanism for MB degradation was investigated to illustrate the roles of each component during the reaction.

## 2 Materials and methods

### 2.1 Materials

Copper (II) acetate and ammonium molybdate were obtained from Macklin Biochemical Co., Ltd. Tetraethyl orthosilicate (TEOS) and polyethylene glycol 600 (PEG-600) were purchased from Aladdin Industrial Corporation. Deionized water was purified using Milli-Q system (Millipore Co., United States). Absolute ethyl alcohol and 30% hydrogen peroxide were sourced from Sinopharm Chemical Reagent Co., Ltd. Phosphate buffer saline (PBS) was sourced from Hyclone. All the reagents were used without further purification.

### 2.2 Synthesis of Cu_2_O nanospheres

Cu_2_O was synthesized by a modified method, which was reported in the literatures (Zhang et al., 2011; An et al., 2018). In the procedure for Cu_2_O synthesis, 80 mg of copper acetate and 1 g of poly−(vinylpyrrolidone) (PVP, Mw 10,000) were dissolved in 30.0 mL of glycol at 70°C. After intensive stirring for 2 h, 10.0 mL of sodium hydroxide (2 mol L^−1^) was added into the above mixture. Gradually, the transparent green solution turned blue, indicating the formatifon of copper hydrate. And then 0.5 h latter, 10.0 mL of ascorbic acid (AA) solution (0.15 mol L^−1^) was quickly introduced into the solution to reduce the Cu (Ⅱ) ions. In order to proceed nanocrystal growth, the turbid yellow suspension was kept in the oil bath with stirring for another 0.5 h. And then it was centrifuged at 10000 rpm for 5 min with decanting the top solution. Finally, the precipitate was washed with distilled water for three times. The obtained Cu_2_O nanoparticles were re-dispersed in 5 mL of ethanol for further use.

### 2.3 Synthesis of Cu_2_O@SiO_2_

5 mL of the obtained Cu_2_O ethanol dispersion were dispersed in a solution (57 mL of ethanol/11 mL of water, 0.4 g of PVP) with ultra−sonication. After stirring for 15 min, the as−prepared TEOS ethanol solution (0.15 mL of TEOS, 1.5 mL of ethanol) was added to the above dispersion and stirred for additional 15 min. Then, 1.0 mL of 0.1 M NaOH aqueous solution was added drop−wisely within 5 min at room temperature with stirring. After 14 h, Cu_2_O@SiO_2_ were collected by centrifugation (8000 rpm, 5 min) and washed twice with 1:2 volume ratio of water and ethanol. Finally, the sample was re−dispersed in 5 mL ethanol.

### 2.4 Synthesis of Cu_2_O@SiO_2_/MnO_2_−PEG

The above Cu_2_O@SiO_2_ was dispersed in 75 mL water using sonication. At room temperature, aqueous PEG (Mw: 600, 1.5 mL, 5 mg mL^−1^) was added into the suspension and mixed for 30 min. After the gentle addition of aqueous KMnO_4_ (1.875 mL, 200 μg mL^−1^), the mixture was stirred for 30 min. Subsequently, the mixture was centrifuged at 8000 rpm. The product washed with DI water and ethanol for three times.

### 2.5 Characterization

The morphology and structural characterization were performed on a scanning electron microscope (SEM, Hitachi S-4800, Japan) and a transmission electron microscope (TEM, JEM-2100F, Japan). X-ray diffraction (XRD) was performed on an Empyrean X-ray diffractometer (PANalytical, Netherlands). UV–vis–NIR spectra were recorded from 200 nm to 800 nm on a UV–vis–NIR spectrophotometer (LAMBDA 1050+, PerkinElmer). An ESCALab 250Xi (Thermo Scientific) spectrometer was used to measure X-ray photoelectron spectroscopy (XPS). An INVENIO S spectrophotometer (Bruker) was operated to acquire the Fourier transform infrared (FTIR) spectra. Photoluminescence (PL) spectra (Ex = 490 nm) were collected on FS 5 fluorescence spectrometer (Edinburgh Instruments) equipped with Xe lamp. The Brunauer-Emmett-Teller (BET) surface areas were obtained using the nitrogen adsorption–desorption isotherms determined at the temperature of liquid nitrogen on an automatic analyzer (Autosorb-iQ-MP, Quantachrome, United States). Electron paramagnetic resonance (EPR) spectra were recorded on a Bruker EMXnano.

### 2.6 Photo−Fenton−like catalytic degradation of methylene blue

The photo−Fenton−like catalytic performances were carried out in a 250 mL quartz beaker equipped with recirculation cooler in a light protective box. The visible light was created by a 300 W xenon lamp through an UV−cut off filter ( ≥ 420 nm). Typically, 100 mL, 10 mg L^−1^ of MB dye and 0.015 g of Cu_2_O@SiO_2_/MnO_2_−PEG was loaded in the beaker. After stirring for 30 min to establish the adsorption/desorption equilibrium, the mixture was exposed to the visible light and subsequently added a known concentration of H_2_O_2_. Samples of the reaction mixture were taken every 15 min intervals in the first 2 h, and 30 min intervals in the following 1 h. Batch experiments included catalyst dosage, concentration of MB, effects of ion species. Furthermore, solution pH variation and the types of reactive species were studied. The purpose was to investigate the catalytic activity and degradation mechanism of MB by Cu_2_O@SiO_2_/MnO_2_−PEG.

By using the external syringe−driven filter, the concentration of MB was investigated *via* a PerkinElmer Lambda 1050 + UV−Vis spectrophotometer. The quality of MB was obtained according to the maximum absorbance at a wavelength of 663 nm. The conversion efficiency for MB was calculated from Eq. 1:

$$\%MB\;dye=C_{t}/C_{0}\times 100\%$$ (1)

The degradation reaction rate constant k was calculated according to the pseudo-first-order degradation reaction using Eq. 2:

$$C_{t\;}{=C}_{0}e^{-kt}$$ (2)

in which C_0_ and C_t_ are the concentrations of MB before and after exposure for a time duration “t”, respectively.

## 3 Results and discussion

### 3.1 Characterization of prepared Cu_2_O@SiO_2_/MnO_2_−PEG

**FIGURE 1:**
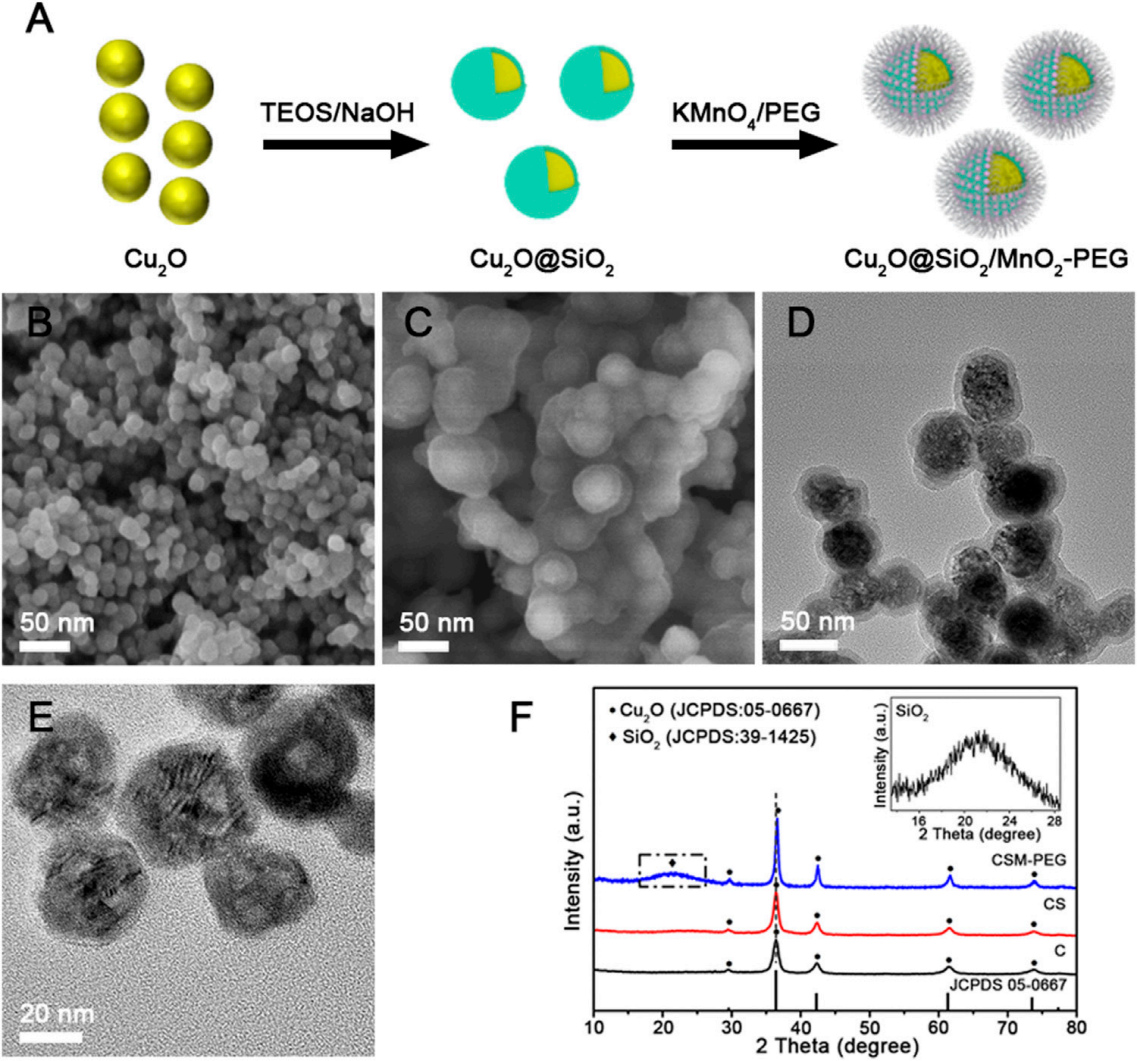
**(A)** The synthesis process of Cu_2_O@SiO_2_/MnO_2_−PEG. **(B,C)** SEM and TEM images of Cu_2_O. **(D,E)** SEM and TEM images of Cu_2_O@SiO_2_/MnO_2_−PEG. **(F)** XRD patterns for Cu_2_O (C), Cu_2_O@SiO_2_ (CS), and Cu_2_O@SiO_2_/MnO_2_−PEG (CSM−PEG). Insert: XRD patterns for SiO_2_.

The synthesis process of Cu_2_O@SiO_2_/MnO_2_−PEG was shown in Figure 1A. SEM and TEM tests further studied the morphology of pure Cu_2_O and Cu_2_O@SiO_2_/MnO_2_−PEG. As shown in Figures 1B,E, Cu_2_O displayed a nearly spherical structure with a diameter of approximately 40 nm. Supplementary Figure S1 gives the size distribution histogram of Cu_2_O in the supporting information. Notably, Figure 1C,D clearly show the core−shell Cu_2_O@SiO_2_/MnO_2_−PEG nanostructure with a 7 nm thick uniform SiO_2_ shell on the Cu_2_O. The existence of such a dense SiO_2_ coating protects Cu_2_O from instability. According to SEM and TEM images, the Cu_2_O@SiO_2_/MnO_2_−PEG particles have diameters of approximately 52 nm. Moreover, Figure 1F shows the X−ray diffraction (XRD) patterns, in which the XRD for pure Cu_2_O (marked as “C”) agreed with standard Cu_2_O (JCPDS card no. 05–0667). Both Cu_2_O@SiO_2_ (marked as “CS”) and Cu_2_O@SiO_2_/MnO_2_−PEG (marked as “CSM−PEG”) exhibit a broad peak due to amorphous SiO_2_ with a characteristic peak at 21.4°, which matched the standard SiO_2_ (JCPDS card no. 39–1425). Remarkably, due to minimal MnO_2_ loading, it was difficult to verify the exhibition of the MnO_2_ component either in electron microscope images or in the XRD patterns. It reveals that Cu_2_O was successfully coated by SiO_2_ shell.

**FIGURE 2:**
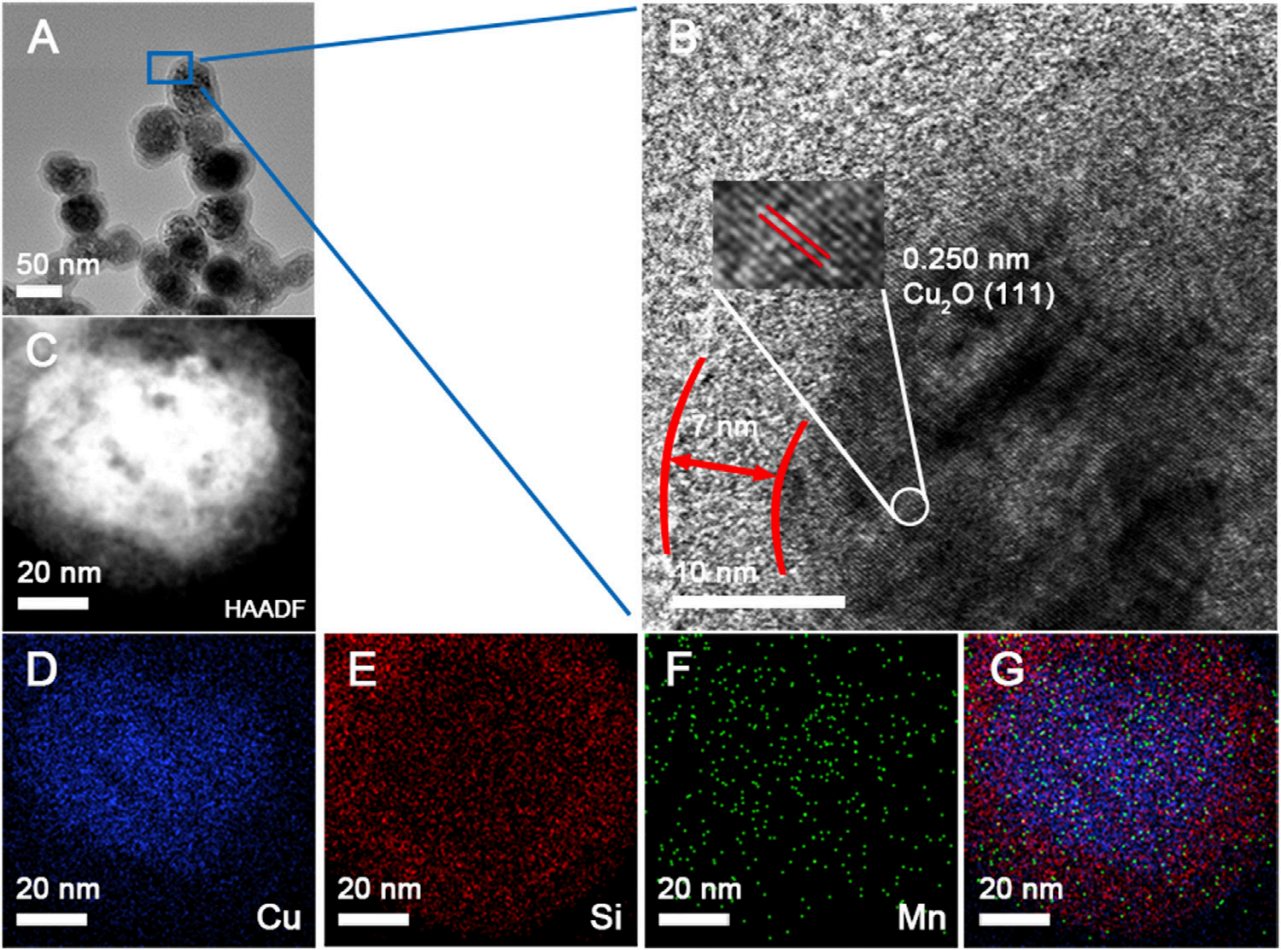
**(A,B)** TEM and HRTEM images of Cu_2_O@SiO_2_/MnO_2_−PEG. **(C−G)** HAADF−STEM image and element mapping of Cu, Si, and Mn, as well as overall maps for the Cu_2_O@SiO_2_/MnO_2_−PEG composite.

As shown in Figures 2A,B, the lattice fringes of Cu_2_O and the interfaces of Cu_2_O and SiO_2_ were resolved clearly in the high−resolution TEM (HRTEM). The set of lattices with interlayer spacings of 0.250 nm corresponded to the (111) planes of Cu_2_O. Furthermore, elemental mapping investigated the homogeneous distribution of Cu, Si, and Mn in the Cu_2_O@SiO_2_/MnO_2_−PEG composite. Figure 2C−G verify that Cu_2_O is coated entirely by SiO_2_ and that MnO_2_ uniformly incorporated throughout the SiO_2_ shell, which suggested successful loading of MnO_2_ on SiO_2_. It is worth mentioning the special core−shell structure enabled the integration of MnO_2_ and unstable Cu_2_O.

**FIGURE 3:**
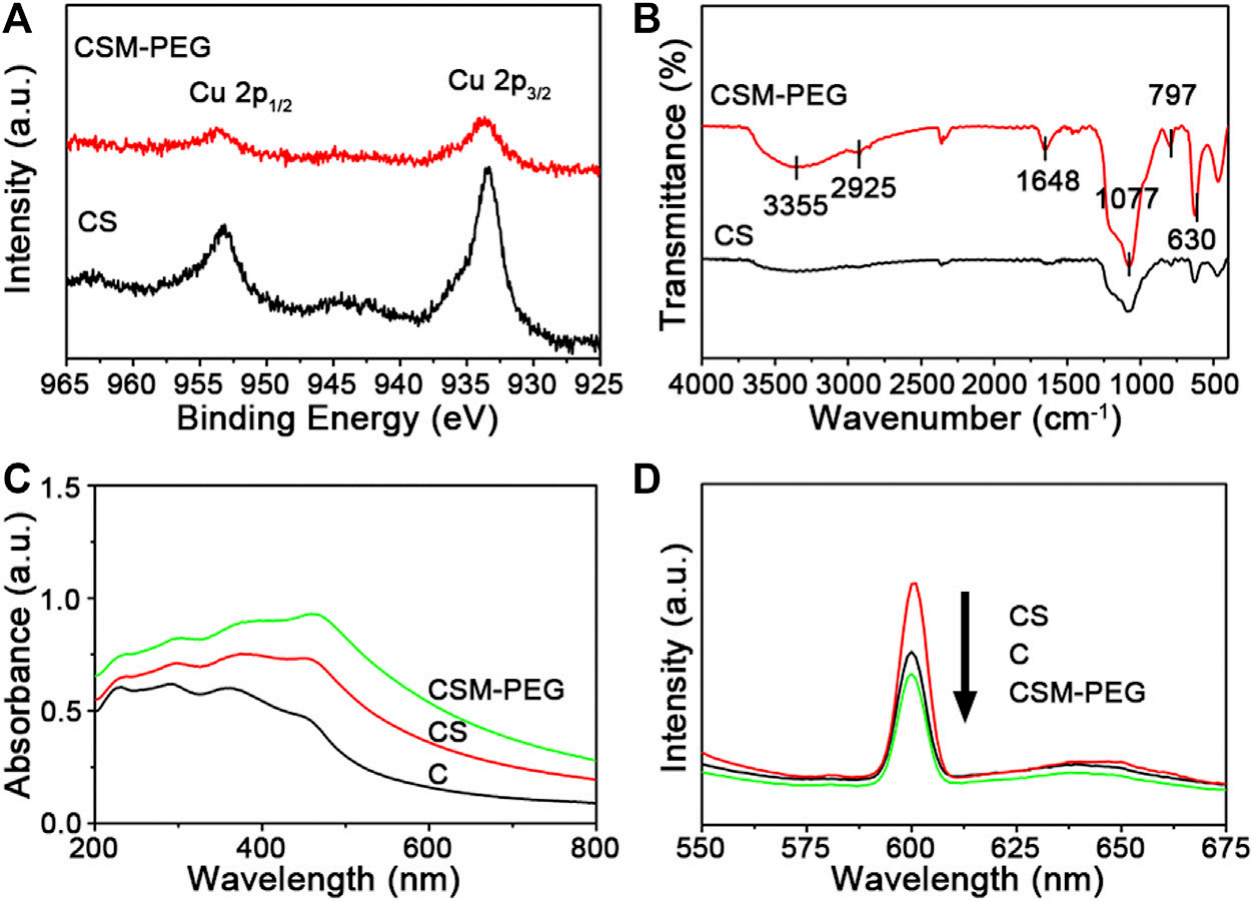
**(A)** Cu 2p XPS spectra for Cu_2_O@SiO_2_ and Cu_2_O@SiO_2_/MnO_2_−PEG. **(B)** FT−IR spectra for Cu_2_O@SiO_2_ and Cu_2_O@SiO_2_/MnO_2_−PEG. **(C)** UV−Vis spectra for Cu_2_O, Cu_2_O@SiO_2,_ and Cu_2_O@SiO_2_/MnO_2_−PEG dispersed in water. **(D)** PL spectra of Cu_2_O, Cu_2_O@SiO_2,_ and Cu_2_O@SiO_2_/MnO_2_−PEG suspension.

X−ray photoelectron spectroscopy (XPS), as a sensitive characterization technique, determined the amount of each element in the sample. Herein, XPS spectra for Cu_2_O@SiO_2_ and Cu_2_O@SiO_2_/MnO_2_−PEG were observed. The Cu 2p spectrum shown in Figure 3A contains Cu 2p_1/2_ and Cu 2p_3/2_. Because of the filled 3 days shells in Cu^+^, no charge transfer between Cu^+^ compounds and metallic Cu occurred. Herein, the absence of strong satellite peaks located at 6 eV and 8 eV above the principal Cu 2p line confirmed Cu(Ⅰ), and ruled out the possibility of a CuO phase in Cu_2_O@SiO_2_ and Cu_2_O@SiO_2_/MnO_2_−PEG (Colón et al., 2006). As a result, Cu_2_O nanoparticles were sufficiently protected by SiO_2_. Moreover, the peaks at 933.2 eV and 933.5 eV correspond to Cu 2p_3/2_ (Ghodselahi et al., 2008). Compared with Cu_2_O@SiO_2_, the characteristic peaks for Cu^+^ in Cu_2_O@SiO_2_/MnO_2_−PEG broadened and attributed to the abundant PEG chain on its surface. As shown in Figure 3B, FTIR peaks observed at 3355 cm^−1^, 2858–2946 cm^−1^, and 1077 cm^−1^ were attributed to characteristic −OH, −CH_2,_ and C−O−C vibrations, respectively. Furthermore, the peak centered at 1648 cm^−1^ was assigned to bending and stretching vibrations of the surface OH groups stemming from PEG modifications on the Cu_2_O@SiO_2_/MnO_2_ surface (Caccamo and Magazù, 2017; Singh et al., 2020; Ghauri et al., 2022). Additionally, the broad absorption peaks at approximately 1063 cm^−1^ and 797 cm^−1^ corresponded to the asymmetric stretching mode of Si−O−Si and Si−O−C bonds and symmetric stretching vibration of Si−O bonds, respectively (Cheng et al., 2022). The band at 630 cm^−1^ was coincident with the optically active lattice vibration of Cu−O in Cu_2_O (Zhang et al., 2009; Hao et al., 2021). As a result, the characteristic peaks observed for PEG, SiO_2_, and Cu_2_O in the FTIR spectra confirmed the successful preparation of PEGylated Cu_2_O@SiO_2_/MnO_2_ and agreed with TEM and XPS data. In addition, wavelengths of visible light range from 770 nm to 350 nm, corresponding photon energy from 1.61 eV to 3.54 eV. However, photon energy band gap of SiO_2_ is approximately 9.3 eV (Weinberg et al., 1979), which is much higher than 3.54 eV, resulting in almost no absorption of visible light. Consequently, it is demonstrated that SiO_2_ coating will not inhibit Cu_2_O from photosensitivity. The efficient photocatalytic activity commonly occurs with the complex generation of electron−hole pairs within a semiconductor. UV−Vis spectra for Cu_2_O, Cu_2_O@SiO_2,_ and Cu_2_O@SiO_2_/MnO_2_−PEG were shown in Figure 3C. The peak intensity at 465 nm increased, followed by the SiO_2_ coating, and MnO_2_−PEG incorporation. The irradiation energy accelerated the generation of electron−hole pairs in Cu_2_O@SiO_2_/MnO_2_−PEG under visible light, which increased photocatalytic efficiency. Photoluminescence (PL) spectra of Cu_2_O, Cu_2_O@SiO_2_, and Cu_2_O@SiO_2_/MnO_2_−PEG investigated the migration and separation efficiency of the photo−generated charge carriers. As shown in Figure 3D, the emission intensity at 600 nm decreased in the following order, Cu_2_O@SiO_2_ > Cu_2_O > Cu_2_O@SiO_2_/MnO_2_−PEG. The weakened emission intensity of Cu_2_O@SiO_2_/MnO_2_−PEG indicated low recombination of the electron−hole pairs in VB and CB due to MnO_2_. Thus, the effective separation of electron−hole pairs improved the photocatalytic properties (He et al., 2017; Hao et al., 2021).

### 3.2 Photocatalytic performance

**FIGURE 4:**
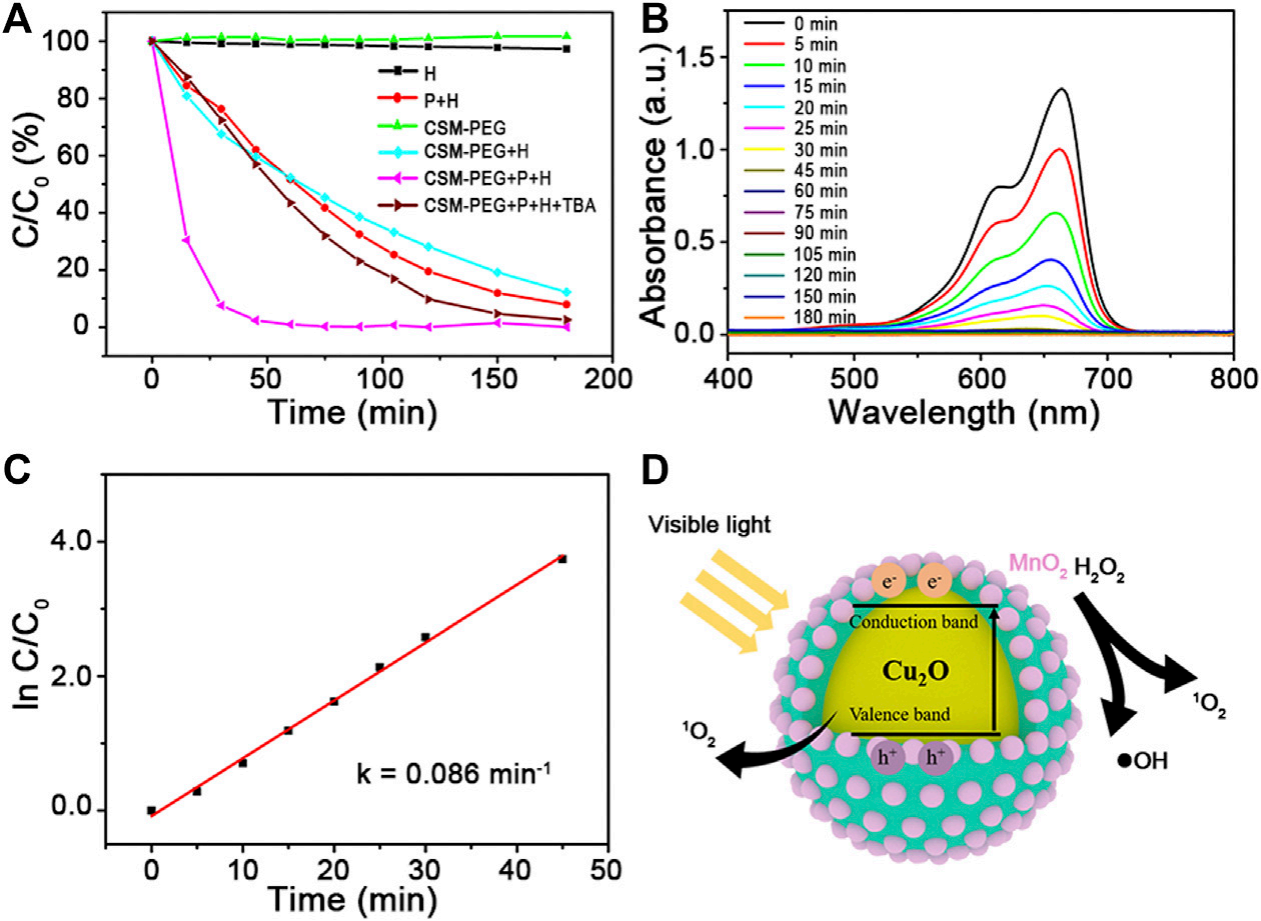
**(A)** Catalytic activities of Cu_2_O@SiO_2_/MnO_2_−PEG for degradation of MB under different conditions. **(B)** UV−Vis spectra measured for the MB solution versus time in the presence of visible light and H_2_O_2._ **(C)** The kinetic fitting lines for H_2_O_2_−enhanced photodegradation of MB over Cu_2_O@SiO_2_/MnO_2_−PEG (k = 0.086 min^−1^). **(D)** Schematic illustration of MB degradation over Cu_2_O@SiO_2_/MnO_2_−PEG under visible light.

To evaluate the photo−Fenton−like catalytic performance of Cu_2_O@SiO_2_/MnO_2_−PEG, degradation of MB was chosen as the model organic pollutant removal reaction. Figure 4A illustrates the degradation efficiencies for MB with Cu_2_O@SiO_2_/MnO_2_−PEG under different conditions, with or without H_2_O_2_, Cu_2_O@SiO_2_/MnO_2_−PEG, and visible light irradiation at wavelengths λ ≥ 420 nm. Virtually no degradation of MB occurred using just H_2_O_2_ or pure Cu_2_O@SiO_2_/MnO_2_−PEG in the absence of light. The concentration of MB decreased by ∼0.3% after an hour, which indicated little catalytic activity for the Cu_2_O@SiO_2_/MnO_2_−PEG catalyst in the absence of an exogenous stimulus. However, Cu_2_O@SiO_2_/MnO_2_−PEG showed effective catalytic activity in the presence of either H_2_O_2_ or visible light and reached MB degradation rates of 47.7% and 34.8% after 60 min, respectively. Subsequently, the catalytic activities of Cu_2_O@SiO_2_/MnO_2_−PEG were investigated with both H_2_O_2_ and visible light irradiation, which resulted in MB degradation rates of 92.5% and 99.0% for 30 and 60 min reaction times, respectively. These results were attributed to MnO_2_ Fenton−like catalytic and Cu_2_O photocatalytic properties, through which reactive oxygen species (ROS) and electron−hole pairs were produced as the active reaction species, respectively (Watts et al., 2005; Furman et al., 2009). Furthermore, H_2_O_2_ acts as a good radical •OH generator because of its advantages in electron acceptor and facilitation in the separation of electron−hole pairs (Zhai et al., 2013). As a result, the integration of MnO_2_ and Cu_2_O promotes MB degradation efficiency under visible light and the presence of H_2_O_2_. Additionally, zeta potential of Cu_2_O@SiO_2_/MnO_2_−PEG before and after adsorption equilibrium were −18.3 eV and −16.4 eV, respectively. No obvious change was observed.

Time−dependent UV−Vis absorption spectra measured MB solution concentrations using Cu_2_O@SiO_2_/MnO_2_−PEG (see Figure 4B). The intensity of the MB absorption peak at 664 nm declined significantly within the first 30 min. This behavior follows pseudo−first−order kinetics, and the value of the linear slope equals the degradation rate constant (Siddiqui et al., 2020). A plot of ln C/C_0_ versus time (see Figure 4C) yielded an MB degradation rate constant of 0.086 min^−1^. In comparison with previous studies, see Table 1, Cu_2_O@SiO_2_/MnO_2_−PEG showed excellent catalytic activity at a sample concentration of 150 mg L^−1^ for MB degradation under visible light with H_2_O_2_ addition. In addition, it is generally accepted that surface areas play a significant role in the photocatalytic activity of photocatalyst. For Cu_2_O@SiO_2_/MnO_2_−PEG, the specific surface areas is 26.8 m^2^ g^−1^, which was much higher than reported Cu−BiVO_4_ (6.5 m^2^ g^−1^). In addition, N_2_ physical adsorption and desorption curves showed typical type IV isotherms with H3-type hysteresis loops, indicating the presence of slit-shaped mesopores formed by the stacking of nanoparticles (supporting information Supplementary Figure S2) (Xu et al., 2020). High surface areas would favor the initial adsorption of MB and providing additional accessible active sites, which benefit to enhance the activity of photocatalyst (Wang et al., 2017b). The schematic illustration of MB degradation under visible light over Cu_2_O@SiO_2_/MnO_2_−PEG was shown in Figure 4D. In addition, the effects of reaction conditions on catalytic activity and possible mechanism of this reaction had been investigated and illustrated carefully in the following study.

**TABLE 1:** Catalytic performances of catalytic degradation of MB dye reported elsewhere.

Catalyst	Sample concentration (mg L^−1^)	Reaction conditions (MB concentration/mg L^−1^; reaction time/min)	Exogenous conditions	Rate constant (10^–3^ min^−1^)	References
ZnO nanostructure	5	1.2; 150	UV light and H_2_O_2_	17.0	Mohammadzadeh et al. (2020)
TiO_2_/micro−cellulose	200	200; 150	Sunlight and H_2_O_2_	29.7	Rajagopal et al. (2020)
1−D Cu(tba)_2_(H_2_O)]⋅2H_2_O	2000	10; 90	UV light and H_2_O_2_	22.2	Wang et al. (2020)
ZnFe_2_O_4_/BiVO_4_	1500	40; 120	Visible light and H_2_O_2_	16.8	Cao et al. (2021)
Fe_3_O_4_@NIP	1000	25; 300	H_2_O_2_	9.4	Zhang et al. (2021b)
Hematite nanoplates	500	10; 120	Visible light and H_2_O_2_	4.3	Zong et al. (2021)
C_44_H_68_FeN_3_O_10_	73	60; 180	Visible light and H_2_O_2_	32.2	Dasgupta et al. (2014)
Fe_2_O_3_−TiO_2_/GA	1000	50; 60	UV light and H_2_O_2_	55.8	Tu et al. (2020)
Cu_2_O@SiO_2_/MnO_2_−PEG	150	10; 45	Visible light and H_2_O_2_	86.0	This research

### 3.3 Effects of reaction conditions on the degradation of MB

**FIGURE 5:**
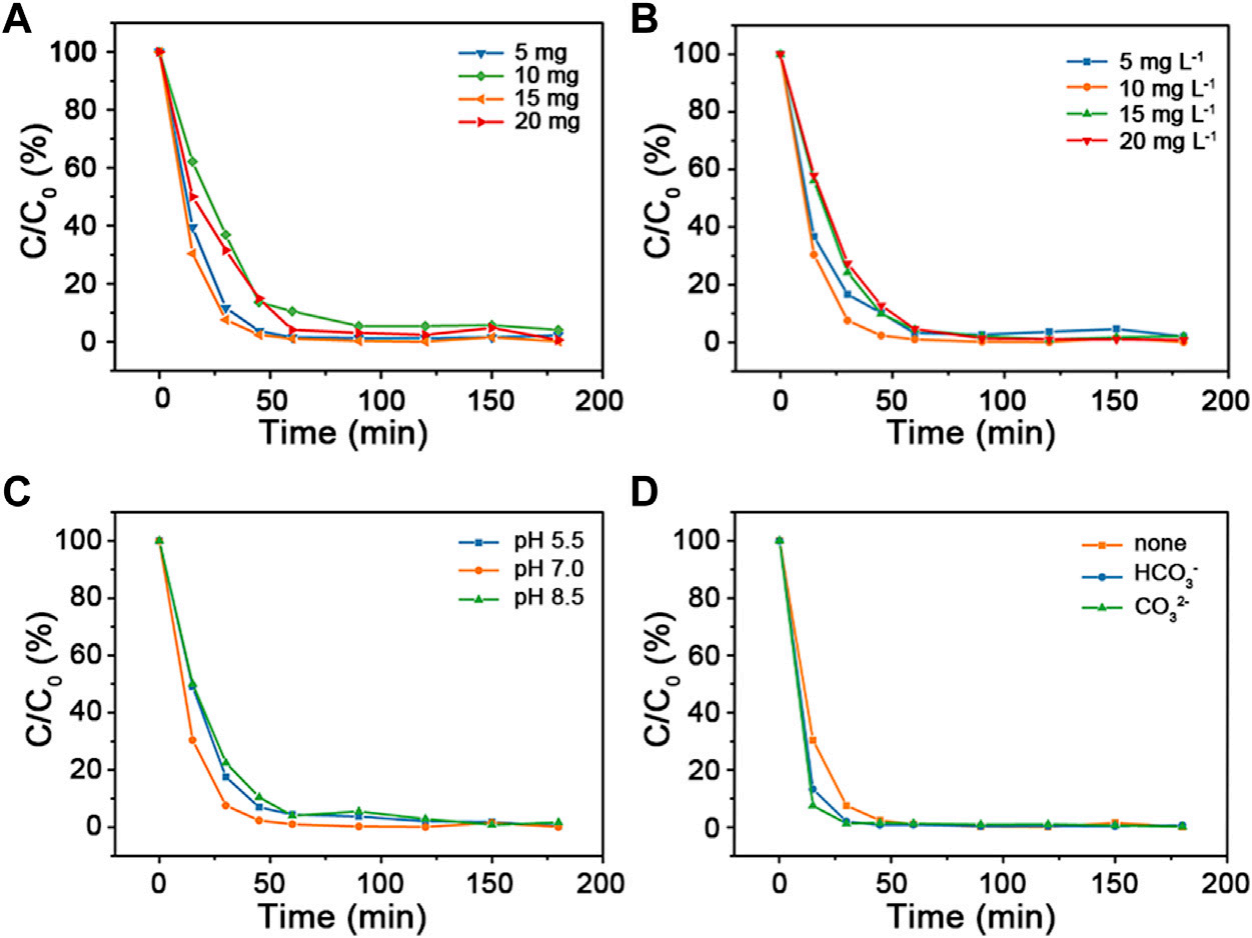
**(A)** Catalytic activities of Cu_2_O@SiO_2_/MnO_2_−PEG for degradation of MB using various catalyst dosage (5 mg, 10 mg, 15 mg, and 20 mg). **(B)** Alteration of initial MB concentration (5 mg L^−1^, 10 mg L^−1^, 15 mg L^−1^, and 20 mg L^−1^) in MB degradation. **(C)** Changes of different pH environment adjusted by HCl and NaOH (pH = 5.5; 7.0, and 8.5) in MB degradation. **(D)** Catalytic performances of MB degradation co-existence of inorganic irons (HCO_3_^−^ or CO_3_^2−^). Common experimental conditions: 15 mg of Cu_2_O@SiO_2_/MnO_2_−PEG; 10 mg L^−1^ of MB concentration; pH = 7.0 of reaction solution without any other inorganic ion.

The influence of reaction conditions (such as catalyst dosage, MB concentration, pH value, and inorganic ions) were carried out. The results were shown in Figure 5. Degradation rate decreases with catalyst dosage increasing from 10 mg to 20 mg. This might be resulted from the agglomeration of Cu_2_O@SiO_2_/MnO_2_−PEG, which resulted in decrement of active points on its surface. However, the degradation rate shows tiny difference for catalyst dosage variation of Cu_2_O@SiO_2_/MnO_2_−PEG. In Figure 5B, with the increasement of MB concentration, the tendency of degradation rate first shows an upward trend and then decreases within 60 min. The 10 mg L^−1^ of MB status presents the highest degradation rate when MB concentration increased from 5 mg L^−1^–20 mg L^−1^. Additionally, the influence of pH on the photodegradation of MB value was evaluated from 5.5 to 8.5. See in Figure 5C, Cu_2_O@SiO_2_/MnO_2_−PEG exibits better photocatalytic activity at pH = 7.0 than others. The pH affected the stability of catalyst, which would further affect the photocatalytic performances. In acid solution, MnO_2_ reacted with H_2_O_2_ to Mn^2+^. Meanwhile, •OH could be scavenged by OH^−^ under alkaline conditions, resulting in less •OH in MB solution (Liu et al., 2015). Therefore, the photocatalytic efficiency decreased with the variation of pH = 7. In fact, various inorganic ions may co-exist in dye wastewater. Herein, the effects of co-existing ions on MB degradation was examined. In current study, HCO_3_^−^ (1500 mg L^−1^) and CO_3_^2−^ (1500 mg L^−1^) were chosen as the extra existence of irons. As shown in Figure 5D, no significant variable is observed in alterative test, which suggests that degradation is well stably controlled. Consequently, the descended removal efficiency would not be caused by the interaction of inorganic irons. As a result, 15 mg of catalyst dosage and 10 mg L^−1^ of MB concentration at pH = 7.0 environment were chosen as the optional experimental conditions. Finally, all of the MB dye was degraded totally.

### 3.4 Mechanism

**FIGURE 6:**
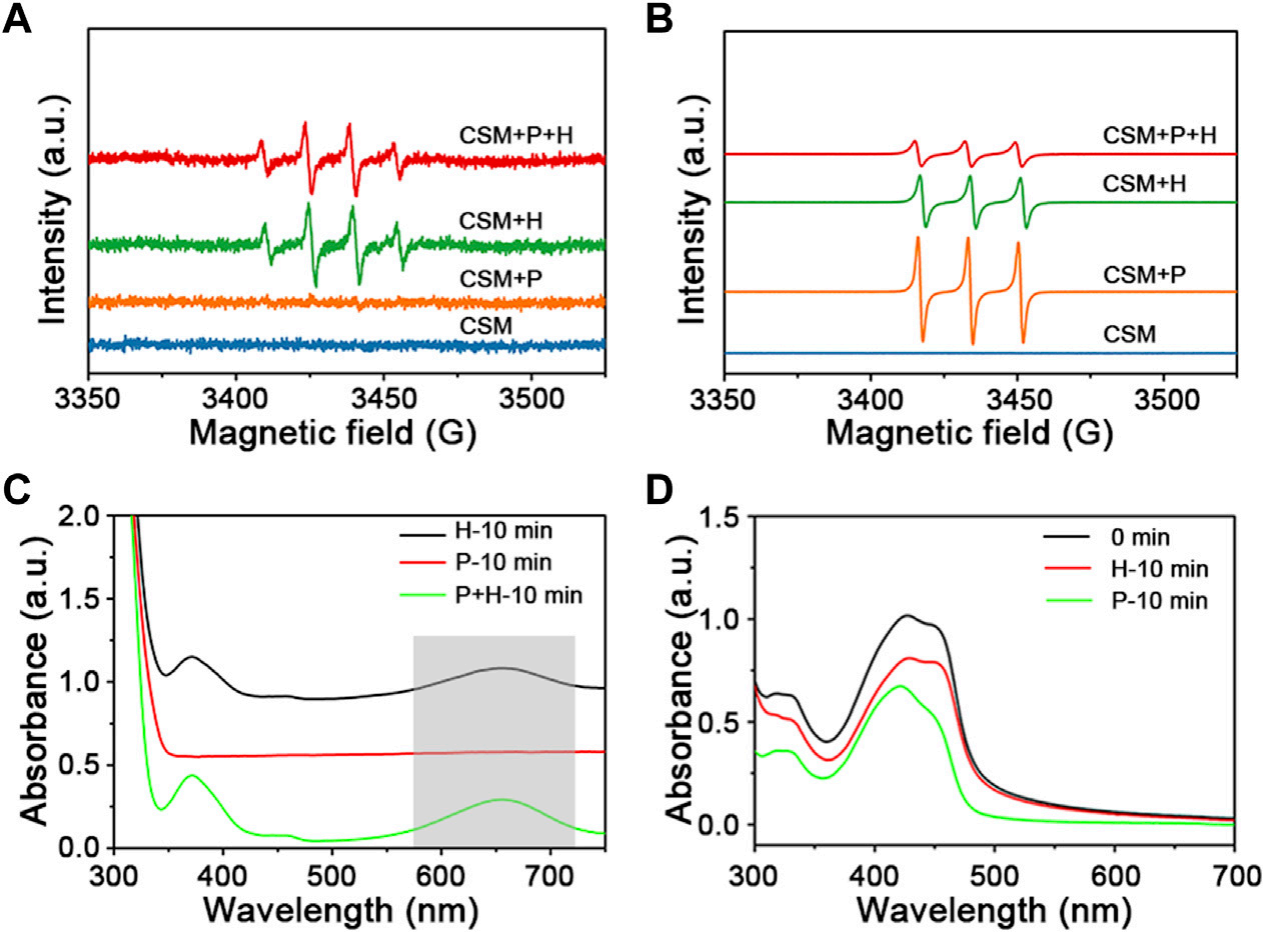
**(A)** EPR spectra of radical adduct trapped by DMPO-•OH with or without H_2_O_2_ (H) and visible light irradiation (P) over Cu_2_O@SiO_2_/MnO_2_−PEG. **(B)** EPR spectra of radical adduct trapped by TEMPO-^1^O_2_. (labelled as “CSM + H″, “CSM + P″, and “CSM + P + H″). **(C)** UV−Vis spectra measuring of TMB in a reaction system with H_2_O_2_ and visible light over Cu_2_O@SiO_2_/MnO_2_−PEG. **(D)** UV−Vis spectra measuring of DPBF with H_2_O_2_ or visible light stimuli.

To investigate the mechanism involved in the photodegradation of MB over Cu_2_O@SiO_2_/MnO_2_−PEG, EPR is employed. 5,5-dimethly-1-pyrroline-Noxide (DMPO, 30 mmol L^−1^) and 2,2,6,6-Tetramethyl-1-piperidinyloxy (TEMPO, 10 mmol L^−1^) were used as the trapping agents of •OH and ^1^O_2_, respectively. It was reported that the strong intensity peaks ratio of 1:2:2:1 and 1:1:1 would be classified to the formation of DMPO−•OH and TEMPO−^1^O_2_, respectively (Harbour et al., 1974; Ma et al., 2022). As shown in Figures 6A,B, there are no signals using Cu_2_O@SiO_2_/MnO_2_−PEG alone. It can be observed that radical •OH was presented with H_2_O_2_ addition instead of visible light exposure over Cu_2_O@SiO_2_/MnO_2_−PEG. Thus, the results indicated the importance role of H_2_O_2_ as the •OH generator. Comparably, ^1^O_2_ was generated in the presence of H_2_O_2_ or visible light. Any of the two ways of stimuli is effective on ^1^O_2_ generation. Finally, the co-stimuli of H_2_O_2_ and visible light may contribute to the high efficient degradation of MB over Cu_2_O@SiO_2_/MnO_2_−PEG.

To confirm the possible mechanism for photo−Fenton−like degradation of MB, an •OH indicator (3,3′,5,5′−tetramethylbenzidine, TMB, 10 mg L^−1^) and an ^1^O_2_ indicator (1,3−diphenylisobenzofuran, DPBF, 2 mg L^−1^) were added. As shown in Figure 6, the appearance of a peak at 652 nm indicated the reaction of •OH and TMB, and the intensity decrease of the peak at 425 nm demonstrated the reaction of ^1^O_2_ and DPBF (Fan et al., 2015; Deng et al., 2019). Radical •OH only was generated during the catalytic decomposition of H_2_O_2_ *via* TMB testing (marked as “H” in Figure 6C). Meanwhile, both visible light and H_2_O_2_ stimuli generated ^1^O_2_, the active species in the catalytic degradation of MB (see Figure 6D). Consequently, the results were exactly consistent with the EPR characterization.

According to the previous evaluation, the possible formation mechanism of main active radicals is as follows. First, owing to short lifetime and limited migration distance of •OH, reactions to generate ^1^O_2_ readily occured (Eq. 3). Furthermore, •OH scavenger t−butanol (TBA, 0.1 g) was introduced into the reaction system, and the time−dependent curve (labeled “CSM−PEG + P + H + TBA”) displayed a similar extension to the “P + H” curve (see Figure 4A). This result agreed with the results shown in Figure 5A, which confirmed the absence of •OH under visible light irradiation (Kang et al., 2019; Yan et al., 2021; Yang L. et al., 2022).

$$4•OH\to^{1}O_{2}+2H_{2}O$$ (3)

Many e^−^ (CB) and h^+^ (VB) pairs formed on the (Cu_2_O) photosensitizer surface *via* visible light irradiation (Eq. 4). At the same time, MnO_2_ catalyzed H_2_O_2_ to generate O_2_, which combined with an electron to form an O_2_^•–^ species (Eqs. 5, 6). Subsequently, O_2_^•–^ generated ^1^O_2_ as shown in Eq. 7 (Zhu et al., 2019; Rajagopal et al., 2020). Therefore, H_2_O_2_ benefited O_2_ production, which resulted in significant amounts of ^1^O_2_. Furthermore, due to PEG modification, many surface OH groups on Cu_2_O@SiO_2_/MnO_2_ acted as hole traps, which facilitated the separation of electron−hole pairs and promoted •OH and ^1^O_2_ production (Trandafilović et al., 2017).

$$Cu_{2}O\overset{\mathrm{hv}}{\to }Cu_{2}O\left(h^{+}+e^{-}\right)$$ (4)

$$H_{2}O_{2}\overset{MnO_{2}}{\to }H_{2}O+O_{2}$$ (5)

$$O_{2}+e^{-}\to O_{2}^{•-}$$ (6)

$$2O_{2}^{•-}+2H_{2}O\to^{1}O_{2}+H_{2}O_{2}+2OH^{-}$$ (7)

$$•OH{/}^{1}O_{2}+MB\to degraded\;products$$ (8)

Ultimately, •OH and ^1^O_2_ triggered the degradation of MB *via* the integration of radical and non−free radical pathways (Eq. 8). Cu_2_O@SiO_2_/MnO_2_−PEG enhanced the catalytic degradation of MB by a photo−Fenton−like process over H_2_O_2_ under visible light irradiation.

## 4 Conclusion

Cu_2_O@SiO_2_/MnO_2_−PEG, with a core−shell structure and an average 52 nm diameter, was synthesized and protected Cu_2_O against oxidation *via* a dense SiO_2_ shell. Integration of Cu_2_O and MnO_2_ united the photo−Fenton−like catalytic properties of Cu_2_O@SiO_2_/MnO_2_−PEG in the presence of H_2_O_2_ under visible light. Additionally, a degradation rate of 92.5% and a rate constant of 0.086 min^−1^ were recorded for MB degradation in 30 min for a 10 mg L^−1^ MB solution, which indicated excellent catalytic activity of Cu_2_O@SiO_2_/MnO_2_−PEG as compared to previous studies. MB degradation was well stably controlled, resulting from no significant change of catalytic performances after catalyst dosage, MB concentration, pH value, and inorganic ions variation. Based on EPR characterization and •OH and ^1^O_2_ indicators, MB degradation proceeds *via* the integration of radical and non−free radical pathways. Thus, Cu_2_O@SiO_2_/MnO_2_−PEG is a promising candidate for the degradation of dye pollutants sensitive to •OH and ^1^O_2_. The growth mechanism in structure−oriented MnO_2_ and its interaction with Cu_2_O requires further exploration.

## Data Availability

The original contributions presented in the study are included in the article/Supplementary Material, further inquiries can be directed to the corresponding authors.
