# Supplementary material for: A facile synthesis of PEGylated Cu2O@SiO2/MnO2 nanocomposite as efficient photo−Fenton−like catalysts for methylene blue treatment
Source: Front Bioeng Biotechnol. 2022 Oct 18;10:1023090. doi: 10.3389/fbioe.2022.1023090 (PMC9623265; doi:10.3389/fbioe.2022.1023090)
Supplement: Supplementary file 1 [file DataSheet1.pdf]

Supporting information

A Facile Synthesis of PEGylated $Cu_2O@SiO_2/MnO_2$ Nanocomposite as Efficient photo−Fenton−like Catalysts for Methylene Blue Treatment

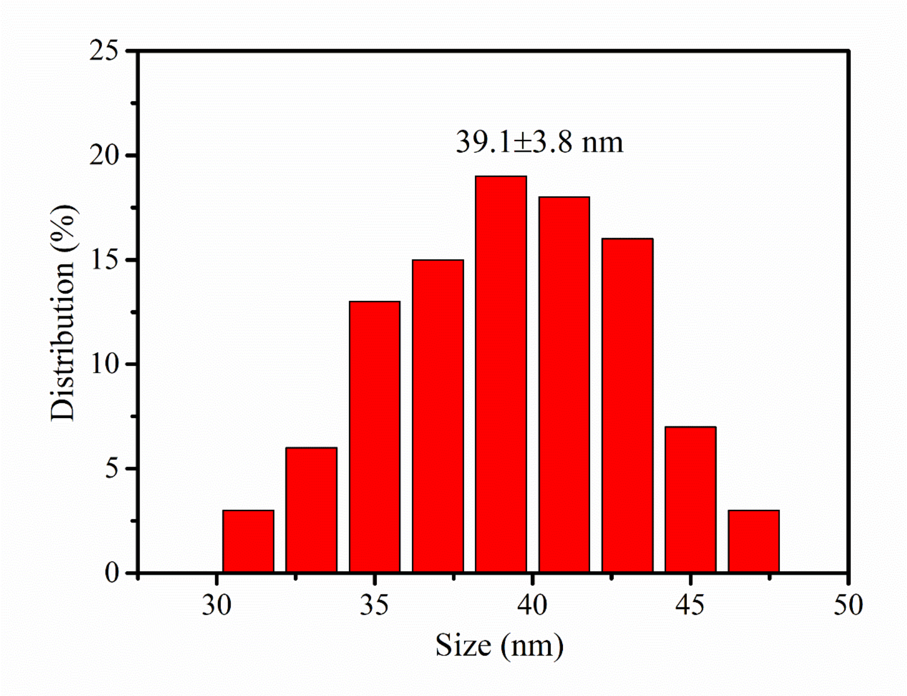

Figure. S1. Size distribution histogram of Cu_2O nanoparticles according to figure 1a.

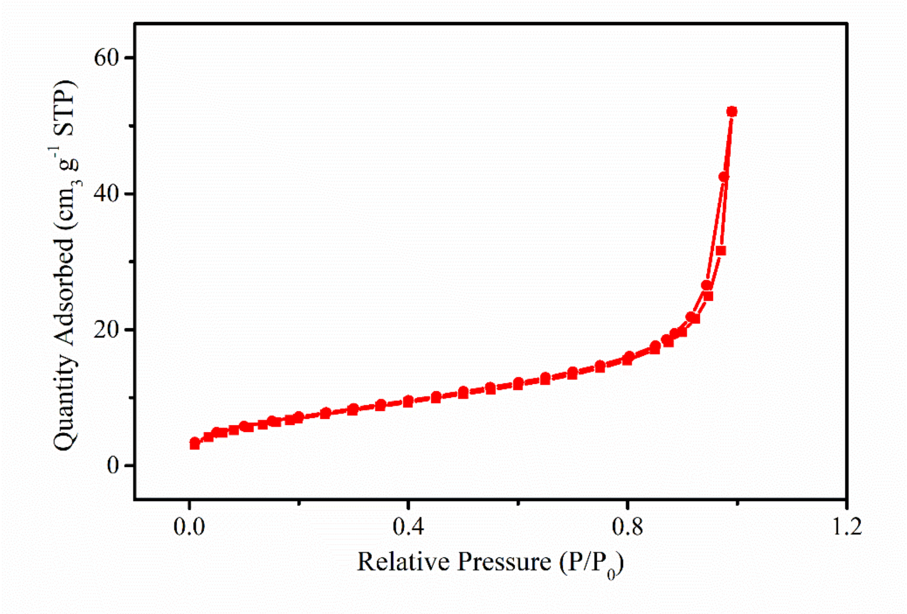

Figure S2. The N_2 adsorption-desorption isotherm curves of $Cu_2O@SiO_2/MnO_2$-PEG.
